# Successive national agricultural policies/programmes, growth of gross domestic product (GDP) and expansion of agribusinesses in Nigeria

**DOI:** 10.1371/journal.pone.0291999

**Published:** 2024-02-21

**Authors:** Clara U. Nwankwo, Michael E. Ikehi, Toochukwu E. Ejiofor, Florence O. Ifeanyieze

**Affiliations:** 1 Department of Agricultural Education, University of Nigeria, Nsukka, Nigeria; 2 Schellhammers Business School, Estepona, Málaga, Spain; Wroclaw University of Environmental and Life Sciences: Uniwersytet Przyrodniczy we Wroclawiu, POLAND

## Abstract

In Sub Saharan Africa, agriculture’s contribution to employment and Gross Domestic Product (GDP) is estimated to be higher than other sectors. Policies designed and implemented for the agricultural sector could be an influencing factor to the variations in the contributions of agriculture to the annual national GDP. These policies are believed to have shaped and (some) still shaping the landscape of agriculture and national economy. The study analysed agriculture’s GDP contribution during the implementation of various national agricultural policies, and the potential of the policies to foster agrobusiness development in Nigeria between 2000 and 2021. The study adopted mixed-method approach. Primary data were collected through a structured questionnaire administered on 29 purposively sampled state Agricultural Development Programme (ADP) directors across Nigeria. The questionnaire was face-validated by three experts. Reliability test was carryout using Cronbach Alpha approach, which yielded an index of 0.89. Copies of the questionnaire were administered on the respondents through direct contact. Secondary data were collected from the Nigeria’s Federal Ministry of Agriculture and Rural Development, National Bureau of Statistics, and World Bank. Data was analysed with mean, standard deviation, percentages and ANOVA. Findings of the study revealed that the performance of implemented agricultural policies had influence on agricultural sector’s percentage contribution to national GDP, and changes in agriculture’s GDP contribution had significant impact on national GDP growth. The duration of active life of the policies did not influence their performance, like the Root and Tuber Expansion Programme which lasted longer yet performed less than the National Special Programme on Food Security in terms of improvement in agriculture’s GDP contributions. All the policies implemented had several limitations in their ability to foster agribusinesses in Nigeria. The study recommends that future policies should focus on providing sustainable frameworks for developing the business in agriculture through value chain optimisation and the use of the teeming, young, and affordable labour force like China and India did to become global food producers.

## Introduction

Agriculture is regarded as a vital part of many countries’ economies, and a major source of livelihood in many regions of the world. As a business for most people, agriculture involves the production of agrarian goods or the engagements in agro-allied value chain activities for the purpose of earning an income or a living [1]. The importance of agriculture to humans and the society includes being a source of food for the people, revenue for governments at various levels, providing raw materials for secondary and tertiary productions, and as a means of livelihood by providing employment for farmers, marketers, and processors of agricultural products and services. Although agriculture produces food and offers opportunities for economic growth, the sector continues to face several challenges, ranging from unstable climatic conditions, weak advisory/extension services, poor diffusion of agricultural innovations, and poor policy design and implementation, mostly in developing countries [2–4].

Policies are designed and implemented to provide direction, control actions–anticipated or in-practice–and solutions to common or specific problems. Policies can be reactive or proactive to a problem and current or futuristic in approach [5]. No matter the type, policies affect every aspect of human and corporate existence, including determining the quality of the air to breathe and water to drink, the food to eat–how it is harvested, where it is distributed and sold, and how much to pay [6,7]. Agricultural policies structure how food systems and related sectors like the environment, and society including the economy operate [8,9]. As agriculture contributes significantly to the economic growth of many Sub-Sahara African (SSA) countries, several governments have sought to improve agribusinesses and food security through policies.

In SSA, Gross Domestic Product (GDP) growth from agriculture is estimated to be higher than any other sector [4,10]. In an emerging economy like Nigeria, agribusiness ventures account for over 70% of labour force employment and contributes over 23% to GDP [11,12]. In other African countries like Sierra Leone agriculture contributes as much as 57.45%, Chad (53.99%), Ethiopia (37.57%), Liberia (36.96%) and Niger (36.48%) to national GDP [12]. At the global level, agriculture still plays significant role in employment and revenue for developed countries. In 2020, China was ranked highest in agricultural production with an annual output valued at US $1.56 trillion, while India at $403.5 billion, The United States at $307.4 billion and Brazil at $135.8 billion followed at a distance close [13]. In the same year, Nigeria’s agricultural output was valued as ₦803 billion (US $2.2 billion at ₦359.2 to a US $ exchange rate).

Nigeria was a leading agro-economy, as the country was among the top producers of palm oil, groundnut, cotton and cocoa globally in the 50’s [14]. However, the discovery of crude oil in the 1950s progressively led to the neglect of the sector, which has seen Nigeria decline in the global market rankings for most agro-produce she was among the leading exporter/supplier–unarguably a resource curse situation. Though global ranking has fallen, Nigeria remains an agrarian country with vast arable land and favourable ecosystem, edaphic and climatic factors that supports a wide variety of crops and animals. To revamp the sector, agricultural policies have been proposed and implemented by successive leaders. Some were designed and implemented as programmes with corresponding structures and personnel to oversee their affairs while others were policies implemented as laws through the national assembly. Example of the policies in the post-colonial era, included Farm Settlement Scheme (1959), National Accelerated Food Production Programme (1972), Operation Feed the Nation (1976), Green Revolution (1980), Agricultural Development Projects (1974), National Economic Empowerment and Development Strategy (1999), among others. These policies and programmes were setup by successive national governments with the intention of improving the welfare of farmers, increasing the contribution of agriculture to GDP, increasing foreign earnings through agriculture, and supporting the economic and business activities in the agricultural sector across Nigeria. These policies lasted different years and had varying impacts on the economy and the agricultural sector.

Since the turn of the 21^st^ century, intensive efforts have been made by successive Nigerian governments to reposition agriculture. Since the year 2000, Nigeria have had five successive presidents; Obasanjo (1999–2007), Yar’Adua (2007–2010), Goodluck (2010–2015) and Buhari (2015–2023) and Tinubu (since 2023). Each of these presidents have had a policy/programme strategy for agriculture. Some of the policies and programmes include Root and Tuber Expansion Programme (RTEP) (2001), National Special Programme on Food Security (NSPFS) (2002), National Food Sector Plan (NFSP) (2007), Agriculture Transformation Agenda (ATA) (2013), Agricultural Promotion Policy (APP) (2016) and National Agricultural Technology and Innovation Policy (NATIP, 2021–2025) [14–19]. These policies at inception had the country’s best interest–following the rational choice believe–but are usually poorly implemented by either the proposing administration or the successor in addition to other limiting factors [14,20–30].

## Theoretical framework

Developed countries that have advanced their agricultural sector through the adoption of robust policies focused on maximizing their available resources and putting into operations some institutional, technological and innovations frameworks to drive the solutions to the issues in the agricultural sector [18]. These approaches when adopted, presumably, favour the arguments presented in rational choice theory. According to Bridge [31,32] Rational Choice Theory (RCT) presents an approach “that seeks to explain human affairs by making certain simplifying assumptions about what motivates their actions”. As first presented by Ronald V. Clarke and Derek B. Cornish in 1986 [33] and described by economist Renzetti [34], RCT “takes as its starting point the principle that humans are rational beings who exercise free will in deciding on a course of action” to solve a problem. Designing and implementing policies are considered as governments’ course of actions (rational choice) for improving and developing important sectors of the economy. The need to improve the agricultural sector in several countries has seen the implementation of potential profitable policies and programmes.

The policy choices to drive the opportunities for improving the situation in agriculture could be focused on the introduction of advanced technologies, creation of intersectoral engagements to improve the agricultural value chain, and implementation of business-driven policies in the sector. Policies focused on improving business opportunities in the agricultural sector are vital and are the way forward [35]. Indicators of improvements in the agricultural sector can be measured–singly or combined–by GDP contribution, export ratio, consumption value, added value and land utilization [36]. This study adopts the measure of agricultural sector’s GDP contribution to assess the performance of the various agricultural policies. Furthermore, the study evaluates the policies on their potential to encourage agrobusiness value chain development.

**Fig 1:**
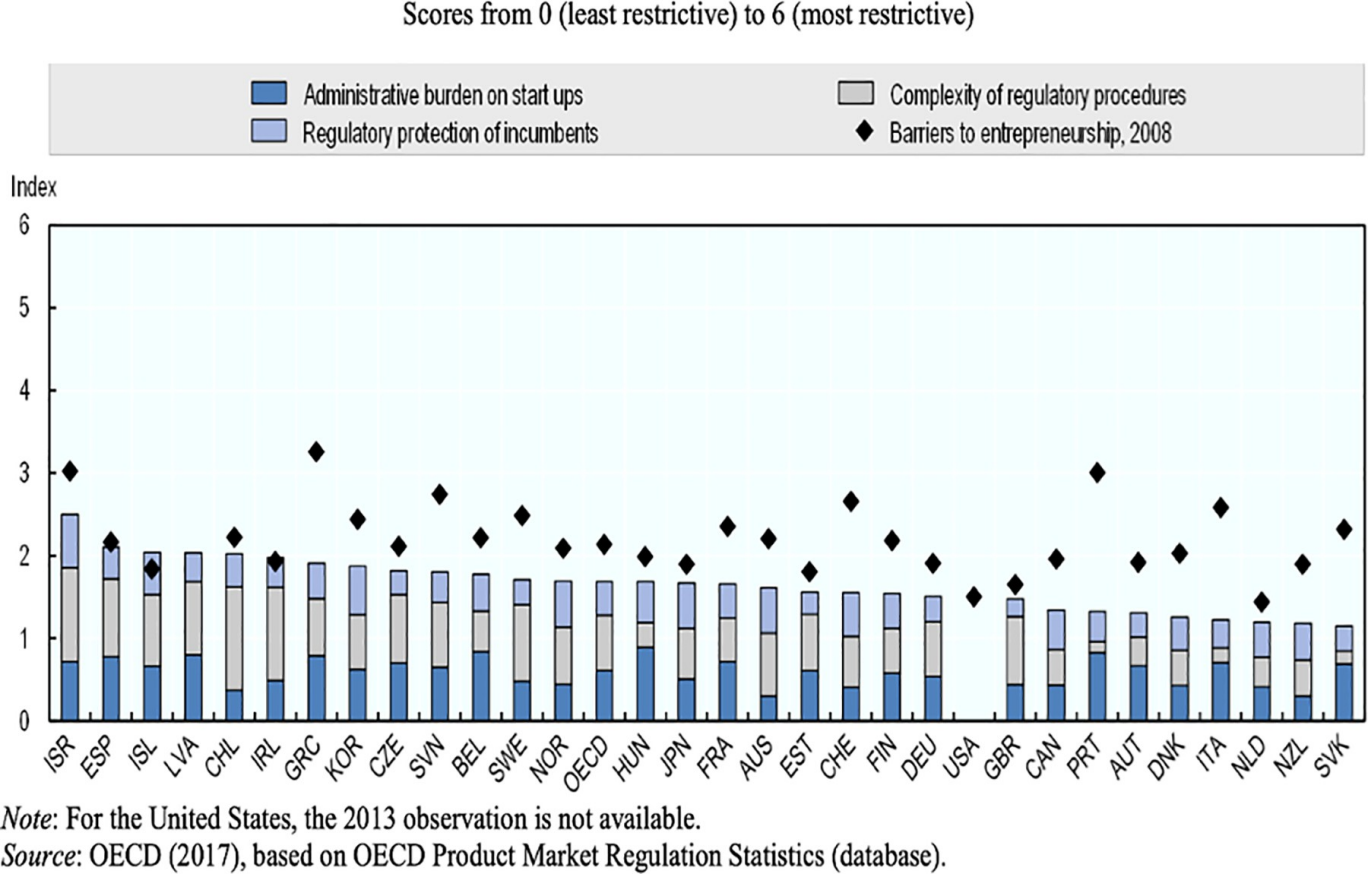
Countries and their ratings on the OECD product market regulation statistics.

According to Organization for Economic Co-operation and Development (OECD) [37] policies that foster the proliferation of businesses mostly focus on lowering entrance barriers for young businesses. For young entrepreneurs, regulatory divergence across countries can impose additional layers of difficulties. Recently, however, across most OECD countries, regulatory barriers to entrepreneurship have been declining (see Fig 1).

In the OECD area between 2008 and 2013, the number of days required to start a business was reduced from 14 to 6, and the cost from 5% to 2% of income per capita. In the last decade, reforms have focused on reducing the administrative burdens on start-ups, lowering legal barriers to entry, and decreasing the costs for regulatory compliance in different areas such as environment, labour legislation, product standards and certification. In Portugal, a policy reform was introduced in 2005 which reduced the time to incorporate a company from several months to as little as one hour; and the fees from EUR 2,000 to less than EUR 400. In Chile, since 2013, a virtual one-stop shop allows the creation of a firm in one day, with a single-step, minimal red-tape and at zero cost. The study follows the analogy of OECD [37] to analyse the agricultural policies implemented in Nigeria in the 21^st^ century on their potentials of encouraging agrobusinesses.

Young entrepreneurs, who consider venturing into agribusiness often cite challenges including underdeveloped value chain, less inclusive policies, high index score for difficulty in doing business, and recently insecurity [37–39]. Poor policy and programme implementation could be a major setback to improving agriculture in developing countries like Nigeria. While several literatures are available on the overall successes and failures of agricultural policies and programmes in Nigeria [23,25,26,39–41], this study assesses these policies and programmes focusing on their impacts on improving agriculture’s GDP contribution and agro-business development in Nigeria. This aspect is missing in available literature and could explain the possible failure and inability of successive policies by successive governments to create sustainable business support structure through policies and programmes for agro-entrepreneurs in Nigeria. The successive development and implementation of agricultural policies by several governments point to the fact that the government recognize the role and significance of agriculture to the economy, and that previous policies have not fully addressed the issues in agriculture or that the problems are dynamic needing continuous policy reforms. Analysing these policies could provide vital information needed for policy reform, formulation, implementation and evaluation and could provide understanding of the strengths and weakness of the policies in promoting the business potentials of agriculture in Nigeria. This study evaluates the relationship between agriculture’s GDP percentage contribution as influenced by implemented policies and annual nation GDP growth in Nigeria. The study further analysed the potentials of the implemented policies to foster businesses in the agricultural sector. Specifically, the study addressed the following research questions:

1. What is the perception of stakeholders on the performance of successive agricultural policies in Nigeria?
2. What was agriculture’s GDP contribution during the implementation of the respective 21^st^ century agricultural policies/programmes?
3. Did the duration of implementation (active life) of the policies influence agriculture’s GDP contribution?
4. Were there potentials for the policies to foster businesses in agriculture?

### Hypothetical presumptions;

- Agriculture’s annual GDP contributions had significant influence on annual national GDP percentage growth rate between 2000 and 2021 in Nigeria.
- Agriculture’s annual GDP contributions had significant influence on annual national GDP between 2000 and 2021 in Nigeria.

## Methodology

The study adopted the mixed-method research approach, relying on both primary and secondary datasets.

### Animal or human consent declarations

The inclusion of humans as respondents for this study received approval from the Departmental Ethics Committee of the Department of Agricultural Education, University of Nigeria, Nsukka.

### Quantitative data

The study collected primary data through a survey carried out in Nigeria. Nigeria is made up of 36 states and each state has a state ministry of agriculture with a state director in charge of the Agricultural Development Programme (ADP). All national agricultural policies/programmes are implemented at the state levels through the ADP. The ADP–which houses the extension service–serves as the bridge between the top stakeholders (policy makers) and the bottom stakeholders (farmers/agrobusiness owners) in the agricultural sector. Therefore, the 36 ADP directors were purposively selected as respondents for this study. A structured questionnaire was used for data collection. The questionnaire had two sections. Section A had 15 items, and each item were assigned four response options of Strongly Agree (SA), Agree (A), Disagree (D) and Strongly Disagree (SD) and were weighted at 4, 3, 2, and 1 respectively. Section B had 5 items with Yes and No response options. The questionnaire was face validated by three experts contacted on ResearchGate. A pilot study was carried out and the data were analysed using Cronbach Alpha approach to establish the reliability and internal consistency of the items on the instrument, which yielded an index value of 0.89. Copies of the questionnaire were administered on the respondents through direct contact. Of the 36 distributed copies of the questionnaire, only 29 were returned and duly completed for inclusion in data analyses.

### Qualitative data

Secondary data were collected from the Nigeria’s Federal Ministry of Agriculture and Rural Development (FMARD), Nigeria’s National Bureau of Statistics (NBS) and the World Bank. Specifically, data on the characteristics of successive implemented policies/programmes were collected from reports in FMARD. Data on GDP contributions of agriculture to national economy between 2000 and 2021 and annual nation GDP growth was obtained from NBS and the World Bank. Assuming other factors to be normal, the study compared GDP contributions of agriculture during the implementation years of each agricultural policy, as an indicator for its influence on the growth and expansion of agribusinesses in Nigeria [36]. This study focused on recent agricultural policies implemented between 2000–2021 in Nigeria. The focus on recent policies is to analyse policies that are currently shaping the landscape of agriculture and national economy. These policies were recently implemented or concluded and their impacts are still been felt.

### Data analyses

Data generated were keyed into SPSS (v28 IBM Corporation, Armonk, NY, USA) for descriptive and inferential statistical analyses. Simple percentage (%) and mean ($\bar{x}$) were used to analyse the nominally distributed data while standard deviation (SD) was used to analyse the level of disparity in the responses of the respondents. In taking decisions for research question 1, a cut-off point of 2.50 decision rule was set for the mean; thus, any item with a mean value higher or equal to 2.50 was regarded as Agreed (A) while less than 2.50 was regarded as Disagreed (D). For the Standard deviation, a low value means convergence of responses therefore, similar response pattern while higher value means divergence of opinion on the same question among respondents.

For secondary data, thematic analysis of the policies/programmes were performed focusing on their life span, primary objective, agriculture’s GDP contribution and level of provisions for improving businesses in the agricultural value chains. To obtain a single GDP value for evaluating agriculture’s GDP contribution during the active life of each policy, cumulation of the annual GDP values were divided by the number of active years of each policy. To evaluate these policies and programmes on their potentials to foster agribusinesses, the study assesses them on their focus on reducing the administrative burdens on start-ups, lowering legal barriers to entry, and decreasing the costs for regulatory compliance in different areas such as environment, labour legislation, product standards and certification [37]. OECD explained that sectorial policies that will favour young businesses must consider lowering constraints and huddles, especially legal requirements and ensure policy compliances, which young businesses and owners face.

### Testing of hypotheses

Data on agriculture’s annual contribution to GDP, annual GDP growth rate and annual nation GDP values between 2000 and 2021 were tested for relationships. Analysis of variance (ANOVA) was used to test associations between dependent and independent variables at 0.05 level of significance. Associations between agriculture’s annual contribution to GDP and annual GDP growth rate, and between agriculture’s annual contribution to GDP and annual nation GDP were analyses. All statistical tests were considered significant at p < 0.05.

## Results

### Research question one

- What is the perception of stakeholders on the performance of successive agricultural policies in Nigeria

**Fig 2:**
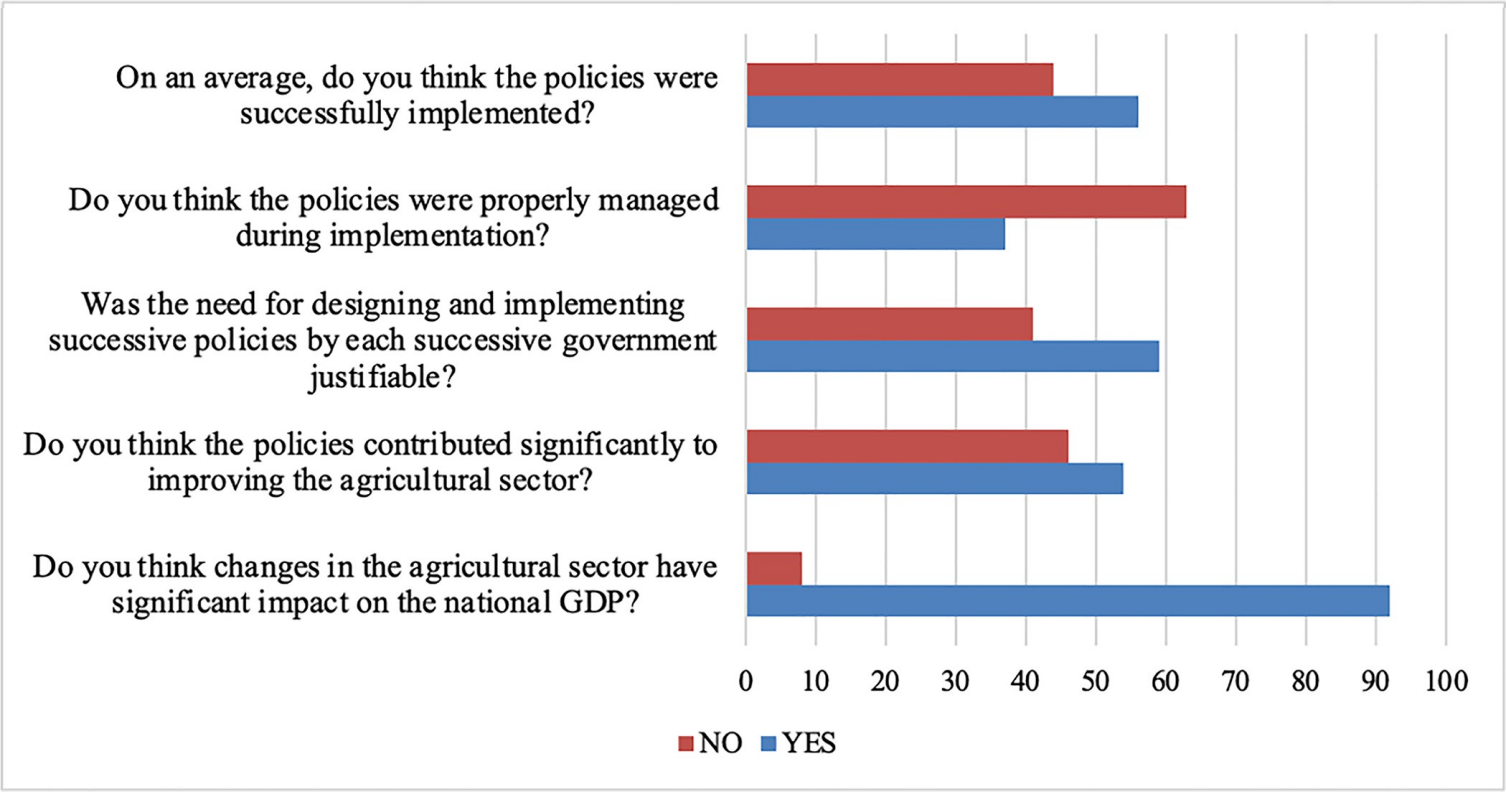
Percentage response of ADP directors on successive implemented agricultural policies in Nigeria.

Data on Table 1 presents the perception of ADP directors on the design and implementation of successive agricultural policies in Nigeria. The respondents agreed to six out of the fifteen presented items while disagreeing to the remaining nine. Standard deviation of all items ranged from 0.31 to 1.21, indicating varied degree of disparities in the opinion of respondents. Fig 2 further explores the perception of the ADP directors on the implemented agricultural programme/policies.

**Table 1:** Perception of stakeholders on the performance of successive agricultural policies N = 29.

S/N	Item Statements	$\bar{x}$	SD	^#^R
1.	Successive agricultural policies have been relatively successfully in improving the agricultural sector over time	2.78	1.09	A
2.	The need to reposition agricultural sectors led to design and implementation of multiple successive policies	3.21	0.31	A
3.	Successive agricultural policies have been strategic in improving business activities in the agricultural value chain	2.43	0.58	D
4.	Agricultural policies are usually conceived and implemented as the need arises	2.9	0.74	A
5.	Failure of previous policies led to designing and implementing new policies	2.39	0.97	D
6.	Most policies were design to attribute actions to each successive government rather than geared towards solving an actual problem	2.46	0.83	D
7.	All implemented policies contributed equally to the development of the agricultural sector	2.01	0.32	D
8.	Implementation of successive agricultural policies influenced the contribution of agricultural sector to gross domestic product (GDP)	3.03	0.92	A
9.	Changes in the agricultural sector influenced the nation GDP	2.58	0.67	A
10.	Successive policies were designed and implemented to build on the success or correct the short falls of the previous one	2.44	0.81	D
11.	The duration of implemented policies (active life span) influenced their performance in changing the agricultural sector	2.39	1.21	D
12.	The objectives and thrusts of successive implemented policies influenced to a large extent their performance	3.42	0.84	A
13.	Successive implemented policies addressed the administrative burdens for start-ups in the agricultural sector	2.41	0.62	D
14.	Successive implemented policies considered lowering legal barriers to entry	2.21	0.65	D
15.	Successive implemented policies favoured decreasing the costs for regulatory compliance, such as in environment, labour legislation and product standards and certification	2.33	0.63	D

^#^R- Decision rule set at 2.50: $\bar{x}$ ≥ 2.50 = Agreed (A); $\bar{x}$ < 2.50 = Disagreed (D).

*Source*: Nwankwo et al.

### Research question two

- What was Agriculture’s GDP contribution during the implementation of the respective 21^st^ century agricultural policies/programmes?

In Table 2, data showed that RTEP was the longest running agricultural policy while the NSPFS was the best performing agricultural policy in the 21^st^ century in Nigeria with an average GDP of 29.76% contribution by agriculture. The least performing was the ATA policy at an average of 20.9% and happened to be the first agricultural policy in the 21^st^ century to address issues in agriculture in Nigeria by implementing private sector partnership with farmers in rural communities. Four of the policies focused primarily on food production while the remaining two were business oriented. However, the business-oriented policies were the least performing ones compared to the policies that focused primarily on food production. There was no substantive agricultural policy between 2011 and 2012.

**Table 2:** Agricultural policies and their percentage GDP contributions.

S/N	Policies	Tenure	Core objective	Policy thrust	Avg. % GDP*	Remark
1	RTEP	2001–2010	Food security through improved production	Social development	26.85	Production focused
2	NSPFS	2002–2006	Increasing food production and eliminate rural poverty	Loan provision	29.76	Production focused
3	NFSP	2007–2010	Increasing food production by intensify land use and irrigation systems	Rehabilitation and expansion of irrigation infrastructure	25.15	Production focused
4	ATA	2013–2019	Agriculture centred on business-like attitude driven by the private sector	Increase access to funding through loans	20.9	Business focused
5	APP	2016–2020	Build an agribusiness economy capable of delivering sustained prosperity	Productivity through private sector partnership	21.8	Business oriented production
6	NATIP	2021–2025	Improved inputs, improve the linkage between agricultural research and training institutions	Commodity value chain improvement	25.36	Production focused

GDP

*—arrived at Cumulative GDP values for agriculture from implementation to phase-out year by the number of years of active life of the policy. See Appendix B.

*Source*: Nwankwo et al.

### Research question three

**Fig 3:**
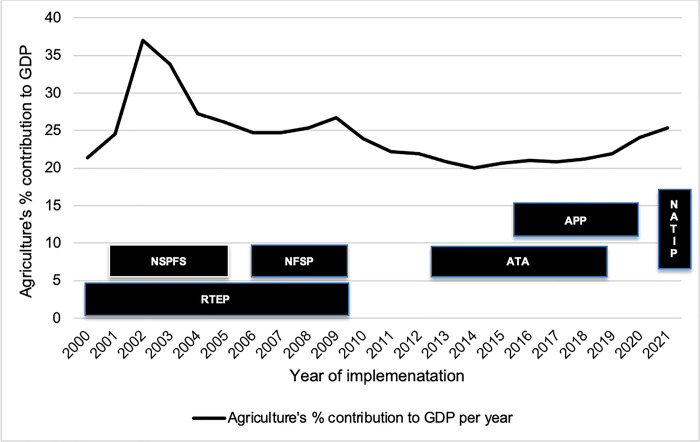
Policies’ duration of implementation and agriculture’s contribution to GDP.

The Fig 3 shows the timeline of the policies and their corresponding percentage GDP contribution to the national economy within their years of implementation. The longest lasting policy was RTEP, from 2001 to 2010. Its first year of implementation between 2001 and 2002 saw a dramatic rise of agriculture’s GDP percentage contribution which invariably favoured the NSPFS policy. The phasing out of the RTEP alongside the NFSP policies in 2011 saw the continued decline of agriculture’s GDP percentage contribution until 2019 where a slow climb was noticed. The missing bar between 2011 and 2012 means there was no agricultural policy in Nigeria to report and analyse. The lack of active agricultural policy within the period probably facilitated the decline of agriculture’s contribution to GDP in the same period, as seen on the chart–Fig 3. This buttresses the importance of an active national policy in the sector.

### Research question four

- Were there potentials for fostering the business in agriculture for each policy/programme?

The data on Table 3 shows the analyses of the potentials of the implemented policies to foster the business in agriculture by reducing the constraints and huddles for new or young agribusinesses as suggested by OECD [37]. The RTEP, NSPFS and NFSP policies mostly focused on decreasing the costs for regulatory compliance for agricultural production but neglected the potentials of reducing the administrative burdens on start-ups, lowering legal barriers to entry and decreasing the costs for regulatory compliance relating to environment and labour registration. These policies focused on increasing the quantity of food produced at rural farming communities. The strategy employed to achieve these policies were to provide loans and improved planting materials for farmers. Data on Table 3 further revealed the potentials of ATA, APP and NATIP to foster the business in agriculture. The ATA by design and implementation offers the potential for reducing the administrative burdens on start-ups and decreasing the costs for regulatory compliance in labour legislation and product standards. The APP offers the potential for reducing the administrative burdens on start-ups, lowering legal barriers to entry and decreasing the costs for regulatory compliance in production. The NATIP policy offers the potential for decreasing the costs for regulatory compliance in environment, labour and production. The policies were not comprehensive enough as none of the policies possessed the complete requirements to ensure business expansion in agriculture.

**Table 3:** Agricultural policy’s’ potential to foster agribusiness in Nigeria.

S/N	Policies	Potential to reduce constraints and huddles				
		Reducing the administrative burdens on start-ups	Lowering legal barriers to entry	Decreasing the costs for regulatory compliance in:		
				Environment	Labor legislation	Product standards and certification
1	RTEP	X	X	X	X	√
2	NSPFS	X	X	X	X	√
3	NFSP	X	X	X	X	√
4	ATA	√	X	X	√	√
5	APP	√	√	X	X	√
6	NATIP	X	X	√	√	√

*Source*: Nwankwo et al.

### Hypotheses testing

In Table 4, at F-value of 18.89 and p-value of 0.0003, agriculture’s annual GDP contributions had significant influence on annual national GDP percentage growth rate between 2000 and 2021. At F-value of 16.51 and p-value of 0.0006, agriculture’s annual GDP contributions had significant influence on annual national GDP value between 2000 and 2021.

**Table 4:** Testing H_01_ and H_02_.

ANOVA							
* *	*df*	*SS*	*MS*	*F*	^*^ *Significance F*	*Remark*	H
Regression	1	142.97	142.97	18.89	0.0003	S	H_01_
Residual	20	151.39	7.57				
Total	21	294.36					
Regression	1	232896.63	232896.63	16.51	0.0006	S	H_02_
Residual	20	282118.63	14105.93				
Total	21	515015.26					

* Significant at p<0.05. S–Significant.

Therefore, the hypotheses, H_01_ and H_02_, were upheld as 0.0003 and 0.0006 critical values of F for H_01_ and H_02_ respectively, were less than the significance value of 0.05. Although Nigeria relies heavily on crude oil, the country remains an agrarian state and agriculture is a key sector in the Nigerian economy as indicated by the upheld hypotheses.

## Discussion

### Perception of stakeholders on the performance of successive agricultural policies in Nigeria

Response of the respondents reflect the perception of an important set of stakeholders in the agricultural sector–directors of ADP in 29 states in Nigeria. The ADP directors play significant role in interacting with the top stakeholders–the policy makers, and the target beneficiaries of implemented policies–the farmers and agrobusiness owners. Most notable in their response pattern on Table 1 are the items they agreed to; items (S/N 1, 2, 4, 8, 9 & 12), especially item S/N 2. The need to reposition the agricultural sector led to the designing and implementation of multiple policies by successive governments (and had the lowest standard deviation value at 0.31, which revealed close convergence of opinions among respondents), as against the believe that policies were design to attribute actions to successive governments–which the respondents disagreed to (see Table 1 S/N 6). The actions of the successive governments fall within the tenets of rational choice theory. The rational choice of designing and implementing agricultural policies by successive governments in Nigeria is presumably the strategic course of action for harnessing opportunities–present and anticipated–in agriculture to achieve the aim of diversifying the economy and improving national welfare. However, the policies seem to fail the OECD test of addressing administrative burdens, lowering legal barriers to entry and decreasing the costs for regulatory compliance for start-ups, as revealed by the response of the ADP directors (see Table 1 S/N 13–15). The failure of the policies has been attributed to several factors [14,19,26–28] including poor management of the policies post-implementation as indicated by the response of the ADP directors (see Fig 1 item 2).

Like China, the giant of Asia and a formidable world power, Nigeria is acclaimed the giant of Africa with respect to population and other classifications. However, unlike Nigeria, China and India have been able to use their massive population–which offers cheap, affordable and readily available labour force–to improve their agricultural and other sectors. It is reported that “China has only 10% of the world’s arable land yet produces a quarter of the global grain output and leads globally in the production of cereals, vegetables, meat, poultry, and fishery products” [42]. According to FAO [43] “India produces most of the world’s milk, jute, and pulses” and currently the world’s most populated country at 1,428,627,663 people overtaking China at 1,425,671,352 people [44]. A key factor to India and China’s success in agriculture is their maximization of their large population [45]. With Nigeria as the sixth most populated country at 223,804,632 people [44], arguably, successive agricultural policies have not put in place mechanisms to optimise the use of one of her most abundant resources–young and available labour force, even in the face of other challenges within Nigeria. China and India have achieved such heights in agriculture but not without local and global constraints. While much of China’s territory is considered to be too mountainous or too arid for farming and the continuing loss of farmland to industrial and urban development, much of India’s agriculture is mostly subsistent, dependent on seasonal rainfall and shortcomings in infrastructure and produce distribution systems [45]. Therefore, like China and India, Nigeria can overcome her challenges limiting the agricultural sector through proper policies with efficient mechanisms. On the African continent, focusing on critical issues in agriculture have been able to boost the contribution of agriculture to GDP for countries like Benin (at 29.44%), Central African Republic (30.07%), Guinea-Bissau (30.86%), Comoros (35.51%), Mali (35.69%), Niger (36.48%), Liberia (36.96%), Ethiopia (37.57%), Chad (53.99%) and Sierra Leone (57.45%) while Nigeria remains at 23.36% [12]. Nigeria with her unique challenges in the agricultural sector can still advance in the ranking of food production and improvements in the contribution of agricultural sector to GDP with the right mindset and policy to guide the present and future governments.

### Agricultural policies and their percentage GDP contributions

Data on Table 2 presents the breakdown of the various agricultural policies implemented by different governments in Nigeria since 2001. The RTEP implementation thrust was social development. The government believed that improving the social conditions of root and other crops farmers will result to better performance of the agricultural sector. The government through the policy made planting materials (especially for root and tuber crops) widely available and accessible. The policy focused on production to drive the sector and to attract more farmers (entrepreneurs) to establish new farms and those that will work for the established farmers. Average GDP for the agricultural sector within the 10years implementation period was 26.85%. This GDP contribution was one of the highest among the agricultural policies implemented. However, the GDP performance cannot be attributed solely to the RTEP policy as other policies like the NSPFS and NFSP were also implemented during the active period of RTEP. It is also important to note that the GDP percentage contributions attributed to this and any other agricultural policies may not be the sole factor responsible for the performance. Other social-economic and political factors and issues in other sectors of the economy are usually also involved in the improvements or downturn of the agricultural sector. According to Jaji et al [46] there are several problems responsible for the inability of Nigeria as a nation to achieve self-sufficiency in food production especially in root and tuber crops, one of these problems is the poor development of the business portfolio of agriculture in Nigeria. Nwanyanwu and Okowa [47] explained that a missing business link to RTEP policy reduced the economic activities that were possible through the policy.

The NSPFS had the highest *average* GDP percentage contributions (at 29.76%) among the 6 policies analysed. The thrust of the NSPFS policy was the open access to loans and funding for food production. Old and young farmers were granted loans to cultivate farm lands. The policy focused on increasing food production and lower rural poverty. The NFSP policy focused on maximizing land and irrigation usage for food production. Within the period of implementation, agriculture contributed about 25.15% to GDP on *average*. Though the NSPFS and NFSP policies performed well in terms of % GDP contributions, African Development Fund (2006) in its report indicated that the lack of business structures in the policies limited their potentials to contribute more to the improvement of the agricultural sector during their years of implementation.

The ATA policy, unlike the previous ones focused on treating agriculture as a business, going beyond usual production and funding agenda of other policies. The approaches in the policy implementation were to provide funding for farmers to improve production, and then involve private sector in the agricultural value chain. The private sector was to intervein in the processing of the primary products into secondary and tertiary goods, distribute and market primary and processed products from the farmers [29]. However, the ATA policy implementation period recorded the lowest GDP contribution (at 20.9%) by the agricultural sector among the 6 reviewed ones. The policy underperformed likely because it approached agricultural issues from a new angle. The concept of treating agriculture as a business was relatively new to farmers and the private sector. The drag between the two sectors to harmonize over time likely lead to the poor performance of the policy and eventually poor growth of the agricultural sector during the implementation period. Likely at the point of harmonization, the policy was phased out with the exist of the government that designed and implemented the ATA policy. Cerna [48] criticised the lack of continuation of policies by successive governments as a problem to the fulfilment of policies targets, even in OECD countries and in other developed and developing countries.

Like the ATA, APP focused on extending and sustaining the business concept brought into agriculture as a way of improving the sector. The private sector interaction with agriculture were reinvented and incorporated into food and agricultural production. The APP, as an extension of the ATA policy, assumed that the funds made available during the ATA implementation period had stimulated enough production [41]. Thus, the APP policy focused more attention on the agricultural value chains through private sector partnership. However, the policy did not make significant change to GDP percentage contribution of agriculture during the implementation period. The GDP percentage contribution of agriculture during the APP (at 21.8% on *average*) was about 1% (at 0.9%) better than the previous ATA policy, which is arguably not significant. The two business-focused agricultural policies happen to be the least performing ones in terms of agriculture’s GDP contributions during their implementation years.

NATIP focuses on commodity value chain improvement through improved inputs, and providing linkage between agricultural research and training institutions. The assumptions that the ATA policy stimulated adequate production levels in agriculture leading to the implementation of APP to consolidate on the gains from ATA as stated by the Federal Ministry of Agriculture [49] was likely erroneous and misleading. Not only did the ATA perform poorly as indicated in Table 2, the successive APP underperformed as well. To correct these negative feedbacks, the NATIP policy capitalized on the inclusion of research institutions to provide direction. Hitherto, policies were made by government and her parastatals without consulting research institutions [50]. The poor consultation of research institutions likely contributed to the poor performance of most implemented policies in Nigeria. Policy researches provide empirical evidences, systematic analyses and unbiased assessment results that could be relevant for policy reforms. But the NATIP policy seems to begin on the right track, and evidence of success is already recorded with a GDP percentage contribution by agriculture already at 25.36% within the first-one year of implementation.

The consideration for research institutions in NATIP policy favours AIS for the first time. Agricultural Innovation System (AIS) advances the improvements in agriculture and food production by encouraging interactions among key stake holders. AIS is a network of people and organizations determined to develop novel products, services and processes in agriculture into economic use, alongside the institutions and policies that influence the way various agents relate, exchange and utilize information for the good of agriculture [48,50–54]. In retrospect, almost all the agricultural policies discussed focused mainly on production. For this, policies offered either loans or input materials like improved seeds and irrigation rehabilitation. Others focused on reducing rural poverty by increasing food production. However, these approaches neither solved the issues of rural poverty nor improved food production for the teaming population in Nigeria and beyond. Therefore, the hope in NATIP is its novel approach to solving agricultural problems in Nigeria.

### Agricultural policies’ potential to foster agribusinesses in Nigeria

Data on Table 3 reveals the analyses of the characteristics of the implemented policies’ potential to foster agribusinesses in Nigeria. An easily observable characteristics of most agricultural policies in Nigeria is their focus on increasing production as a tool for fighting rural poverty, though a non-successful approach as the number of poor people in Nigeria (estimated at 95.1 million people)–even in urban areas–continue to rise and hunger level with food insufficiency remains topical. The six policies analysed offered the potential for improving agribusiness through quality and quantitative improvements during and after production. The RTEP, NSPFS and NFSP by their design and implementation structure had limited potentials to reduce administrative burdens on start-ups, lower legal barriers to entry agribusinesses, and decrease the costs of regulatory compliance to the environment and labour. These policies majorly recognized the production aspects of agriculture. They further presented and legitimized the social and cultural perception of agriculture and farming as a means of livelihood. The policies did not focus on helping the farmers to grow and manage their farms like a business, as every successful business goes beyond producing [14,40]. The ATA, APP and NATIP brought about various modifications in the design and implementation of agricultural policies. The ATA had the potential of reducing administrative burdens on start-up in its design; farmers were encouraged to patterner with private sector players and value chain actors for the betterment of the sector. In addition to decreasing cost for regulatory compliance for products through access to funding (loan), the ATA policy also accounted for labour costs through recognition and reward for private sector agribusiness players–entrepreneurs.

The APP had the potentials to reduce the administrative burdens on start-ups. The policy addressed this issue for farmers by providing the enabling and interactive environment for farmers and private sectors to cooperate. With this approach, the farmers focused on producing while the private sector was expected to manage the administrative issues involved in doing business in agriculture. The NATIP policies appears to learn and correct the mistakes of previous policies by enabling research institutions to provide viable data for improving the sector.

Most agricultural policies and programmes conceived and implemented in sub-Sahara Africa focused on food security, rural development and poverty reduction through input provisions. These have only solved an aspect of agribusiness–production, a likely reason why the sector remains underdeveloped and less competitive. The focus on production means more output while the inclusion of the business aspect means sustained creation of demand and supply for produce through the development of the agricultural value chain [39,41,54]. Business, even in agriculture goes beyond production. A company can produce adequate quantity of a good in demand but poor branding, marketing and distribution, and management can lead to declines, negative revenue, bankruptcy and subsequent closure of the business. It is thus important to consider agricultural transformation processes that incorporates business management in policy and practice.

Analysis of the hypotheses indicate that agriculture is a significant contributor to both GDP growth rate and total annual national GDP. Indisputably, agriculture remains an important aspect of national economy and employment in Nigeria and across Africa. This possibly justifies the unending attempts by successive governments in Nigeria to revamp the sector. Also, that successive policies in the sectors have not performed excellently, yet the sector’s contribution to GDP is significant, means that future governments and stakeholders in the country will continue to design, redesign and implement agricultural policies with the hope of finding sustainable solution to issues in the sector. The opinion of the ADP directors on the significance of the agricultural sectoral to economic development in Nigeria re-echoes the importance of continuous adjustments of policies for achieving sustainability in the sector see (Fig 1 items 4 & 5). Therefore, it is paramount that researches continue to explore the gaps in implemented policies for possible redesigns. This is most important as (future) governments will continue to make rational choices towards improving the agricultural sector, especially as Nigeria is intensely striving to diversify her economy, away from the heavy dependence on oil, and agriculture is the focus. “The basic premise of rational choice theory is that the decisions made by individual actors will collectively produce aggregate social behaviour” and benefits–*Wikipedia*.*com*.

## Conclusions and recommendations

In general, all agricultural policies implemented in the 21^st^ century in Nigeria had limitations. Their limitations are their inability to cover every aspect of reducing constraints for entrepreneurs in the agricultural sector as recommended by OECD. However, successive agricultural policies continue to modify the sector. Most farmers in the sector are still regarded as subsistent and continue to practice farming as a means of livelihood, not business. An approach to changing this would be to formulate policies that are both production and business oriented. The study argues that government policies and programme ought to consider not only production but provide directions and enabling factors to turn subsistent agriculture to flourishing enterprises and make the sector competitive enough to attract new players. As explained by the rational choice theory, actions of government should yield collective benefit for all. Developing and implementing business inclined policies and programmes for agriculture could stimulate value chain development of the sector, especially with abundant labour forces as in other highly populated countries like India and China. Therefore, new policies for the agricultural sector should, first, promote the establishment of micro and medium firms that are specialized in agriculture. To ensure successful establishment of these firms, likely by young farmers, agricultural policies, must focus on reducing the administrative burdens on start-up, lowering legal barriers to entry, and decreasing the costs for regulatory compliance for new entrepreneurs in the agricultural business. Future policies could grant task holidays for young agribusiness owners and firms, provide grants or loans with single digit interest rate to registering and running new enterprises in agriculture, make registration of agribusinesses simple and possibly complete all process online, and provide accessible structure for sourcing of farm inputs and export of finished products. Most discussions of agriculture in Africa, particularly Nigeria, are focused on production and distribution. Until the policy approach to resolving problems in agriculture is changed to capture the business aspects of farming, the expansion of agricultural entrepreneurs will remain limited and the sector will continue to struggle to develop and compete at regional and international levels.

## Supporting information

S1 File (XLSX)

S1 Appendix (DOCX)
