# Supplementary material for: Successive national agricultural policies/programmes, growth of gross domestic product (GDP) and expansion of agribusinesses in Nigeria
Source: PLoS One. 2024 Feb 21;19(2):e0291999. doi: 10.1371/journal.pone.0291999 (PMC10880958; doi:10.1371/journal.pone.0291999)
Supplement: S1 Appendix — (DOCX) [file pone.0291999.s002.docx]

**Appendices:**

**Appendix A**

Agriculture’s GDP contributions in Nigeria, 2000-2021

| **Year** | **GDP % agric** | **GDP % growth rate** | **Annual GDP**  **(in Billion USD)** |
| --- | --- | --- | --- |
| 2000 | 21.36 | 5.02 | 69.45 |
| 2001 | 24.48 | 5.92 | 72.8 |
| 2002 | 36.97 | 15.33 | 95.05 |
| 2003 | 33.83 | 7.35 | 104.74 |
| 2004 | 27.23 | 9.25 | 135.76 |
| 2005 | 26.09 | 6.44 | 175.67 |
| 2006 | 24.73 | 6.06 | 238.45 |
| 2007 | 24.66 | 6.59 | 278.26 |
| 2008 | 25.28 | 6.76 | 339.48 |
| 2009 | 26.75 | 8.04 | 295.01 |
| 2010 | 23.89 | 8.01 | 366.99 |
| 2011 | 22.23 | 5.31 | 414.47 |
| 2012 | 21.86 | 4.23 | 463.97 |
| 2013 | 20.76 | 6.67 | 520.12 |
| 2014 | 19.99 | 6.31 | 574.18 |
| 2015 | 20.63 | 2.65 | 493.03 |
| 2016 | 20.98 | -1.62 | 404.65 |
| 2017 | 20.85 | 0.81 | 375.75 |
| 2018 | 21.20 | 1.92 | 421.74 |
| 2019 | 21.91 | 2.21 | 448.12 |
| 2020 | 24.14 | -1.79 | 432.2 |
| 2021 | 23.36 | 3.65 | 440.83 |

*Source*: World bank, 2021.
